# RNA-Sequencing Reveals Biological Networks during Table Grapevine (‘Fujiminori’) Fruit Development

**DOI:** 10.1371/journal.pone.0170571

**Published:** 2017-01-24

**Authors:** Lingfei Shangguan, Qian Mu, Xiang Fang, Kekun Zhang, Haifeng Jia, Xiaoying Li, Yiqun Bao, Jinggui Fang

**Affiliations:** 1 College of Horticulture, Nanjing Agricultural University, Nanjing, Jiangsu, PR China; 2 Shandong Academy of Grape, Jinan, Shandong, PR. China; 3 Institute of Horticulture, Zhejiang Academy of Agricultural Sciences, Hangzhou, Zhejiang, PR China; 4 College of Life Sciences, Nanjing Agricultural University, Nanjing, Jiangsu, PR China; Wuhan Botanical Garden, CHINA

## Abstract

Grapevine berry development is a complex and genetically controlled process, with many morphological, biochemical and physiological changes occurring during the maturation process. Research carried out on grapevine berry development has been mainly concerned with wine grape, while barely focusing on table grape. ‘Fujiminori’ is an important table grapevine cultivar, which is cultivated in most provinces of China. In order to uncover the dynamic networks involved in anthocyanin biosynthesis, cell wall development, lipid metabolism and starch-sugar metabolism in ‘Fujiminori’ fruit, we employed RNA-sequencing (RNA-seq) and analyzed the whole transcriptome of grape berry during development at the expanding period (40 days after full bloom, 40DAF), véraison period (65DAF), and mature period (90DAF). The sequencing depth in each sample was greater than 12×, and the expression level of nearly half of the expressed genes were greater than 1. Moreover, greater than 64% of the clean reads were aligned to the *Vitis vinifera* reference genome, and 5,620, 3,381, and 5,196 differentially expressed genes (DEGs) were identified between different fruit stages, respectively. Results of the analysis of DEGs showed that the most significant changes in various processes occurred from the expanding stage to the véraison stage. The expression patterns of *F3’H* and *F3’5’H* were crucial in determining red or blue color of the fruit skin. The dynamic networks of cell wall development, lipid metabolism and starch-sugar metabolism were also constructed. A total of 4,934 SSR loci were also identified from 4,337 grapevine genes, which may be helpful for the development of phylogenetic analysis in grapevine and other fruit trees. Our work provides the foundation for developmental research of grapevine fruit as well as other non-climacteric fruits.

## Background

Grapevine is one of the most economically important and globally cultivated fruit crops, producing about 77.2 million tons of grape berries in (FAO, 2013), which can either be consumed fresh or processed into juices and liquors [1]. Grape berry growth and development is a complex process displaying a dual sigmoidal pattern with three distinct phases, including two periods of growth intervened by a lag phase characterized by slowing of fruit expansion and maturation of seeds [2]. Phase I is also known as the hard green stage, and is characterized by general cell division and cell enlargement in berries, with a massive concomitant accumulation of amino acids, tannins, and organic acids [3–7]. During phase II, growth of berries slows markedly, organic acid concentration reaches its highest level and sugar accumulation commences [4]. Phase III starts after véraison. During this phase, the growth rate of berries increases rapidly along with their softening, and fundamental changes in metabolites, such as accumulation of sugar and pigments, loss of chlorophyll, etc. [3, 4].

Grapevine crop is classified into two major groups depending upon its consumption and utilization: table grapes (fresh consumption) and processing grapes (wine and raisins). The fruit color, firmness, and sugar content are particularly of great importance to the grape industry. Grape cultivars can be divided into three classes based on fruit color: black, red, and white [8]. Normally, fruit color is mainly determined by the composition and content of anthocyanins [9]. Recently, genetics and genomic studies have revealed key structural genes and transcription factors that are involved in the anthocyanin biosynthesis pathway. *UDP-glucose*:*flavonoid 3-O-glucosyltransferase* (*UFGT*) was identified as the critical factor for anthocyanin biosynthesis in grape berries [10, 11] since its expression was observed only in colored grapes, but not in white grapes. Additionally, the transcription factors *VvMYBA1* and *VvMYBA2* were found to regulate the activity of *UFGT* and also the last steps of anthocyanin pathway [11, 12]. Previous reports have indicated that white-skinned grape berries are homozygous for *VvmybA1a*, while color-skinned berries are either heterozygous with alleles *VvmybA1a*/*VvmybA1b* or *VvmybA1a*/*VvmybA1c*, or are homozygous for *VvmybA1c* [9, 13, 14].

Along with color development, fruit softening is one of the key features of fruit maturity, which is associated with the disassembly of primary cell wall and middle lamella [15]. In grape berries, these processes are particularly attributed to the activity of expansins (Exp), pectin methylesterase (PME), pectate lyase (PL) and xyloglucan endotransglycosylase/hydrolase (XTH) [16–20]. Ishimaru et al. [21] identified three *exp* genes in grape, of which the transcript level of only *Vlexp3* was closely associated with berry softening. Moreover, 29 *exp* genes were isolated from the grapevine genome, but only a few of these genes were associated with fruit development [17]. The enzyme activities of α-galactosidase (EC 3.2.1.22), β-galactosidase (EC 3.2.1.23) and pectin methylesterase (EC 3.1.1.11) were present throughout the process of berry development, while the transcripts for β-galactosidase, pectin methylesterase, polygalacturonase, pectate lyase (EC 4.2.2.2) and xyloglucan endotransglycosylase (EC 2.4.1.207) were also present during berry ripening [19]. Moreover, turgor pressure of mesocarp cell and abscisic acid (ABA) also contributed to fruit softening [22–24].

Sugar accumulation occurs after véraison stage prior to fruit coloring and softening phases [2]. Sugar transporter genes (*VvSUC11* and *VvSUC12*) are expressed mainly after véraison, when sugar concentration increases [25, 26]. Six hexose transporters related to glucose and fructose have been cloned from grape berries and named *VvHT1-6* [27]. Among them, VvHT1 protein was abundant during the early stages of berry development but was absent from ripening berries [28, 29]. *VvHT2* was associated with véraison and was weakly expressed in ripening berries, while the expression level of *VvHT6* was also associated with the véraison stage and was strong during grape maturation [27].

Transcriptome sequencing using NGS technologies has been increasingly carried out in model and non-model plants for gene detection and marker development [30, 31]. To date, RNA-seq technology has been widely used to detect gene expression in grape fruits [32, 33], leaves [34], flowers [35], and in response to different environmental stresses [36, 37]. Interestingly, most of the grapevine transcriptome work focused on wine grape cultivars due to their high economic demand.

In this report, we used the RNA sequencing (RNA-seq) approach to carry out a comprehensive analysis of the global transcriptional profile of table grapevine berry (hybrid of *V*. *vinifera* and *V*. *labrusca*, ‘Fujiminori’) during the expanding period (40DAF), véraison period (65DAF), and mature period (90DAF). We investigated the suitability of a number of reference transcriptomes for RNA-seq analysis in grapevine, validated the number of transcriptional changes observed using quantitative real-time PCR, and described the biological processes during berry development such as fruit color development and softening, lipid metabolism, and sugar accumulation.

## Materials and Methods

### Grape berry sampling and development

Grapevine trees were grown in the fruit experiment station in Nanjing (N32°02’12.77”, E118°37’33.25”), and the experiments were carried out under the supervision and permission of the deans of College of Horticulture. Berries from three year old ‘Fujiminori’ grapevine trees were sampled at the fruit expanding (40DAF or DAF40), véraison (65DAF or DAF65), and ripe (90DAF or DAF90) stages throughout the growing season. Furthermore, in order to capture a representative biological selection of transcripts at each time-point, RNA for Illumina sequencing was purified from tissues of 40 berries sampled from 20 bunches.

### Total RNA extraction and purification, construction of cDNA library and Illumina deep sequencing

Total RNA was extracted from grape fruit samples using TRIzol reagent (Invitrogen, Carlsbad, CA, USA), and the processed RNA was checked for purity and integrity using Nanodrop-2000 spectrophotometer (Thermo Scientific, Wilmington, DE, USA) and Bioanalyzer 2100 (Agilent Technologies, Santa Clara, CA, USA). An mRNA-seq library was constructed using the Ultra™ RNA Library Prep Kit for Illumina (NEB, Ipswich, MA, USA) following the manufacturer’s instructions, and the average size of the cDNA library was 270bp. The samples were sequenced on an Illumina Hiseq^TM^2500 platform. Each sample yielded more than 4GB of data. Sequencing was performed by the Shanghai Hanyu Biotechnology Company (Shanghai, China).

### Differential Gene Expression Analysis

Raw data were filtered by FASTX-toolkit. Clean reads were aligned to the grape genome using TopHat with the default parameters [38]. Following alignment, the count of mapped reads from each sample was derived and normalized to reads per kilobase of exon model per million mapped reads (RPKM). Differential expression was analyzed and calculated based on the count values of each transcript between libraries using edgeR (the Empirical analysis of Digital Gene Expression in R) software [39]. The thresholds for judging significant differences in transcript expression were “FDR < 0.001” and “|log_2_ fold-change (log_2_FC)| ≥1”. Genes with observed RPKM <0.3 were considered as not expressed in the sample and were therefore excluded in at least one group. Venn diagrams were built using the online available tool Venny (http://bioinfogp.cnb.csic.es/tools/venny/) [40].

### Functional annotation

The DEGs between 40DAF and 65DAF fruits, or 65DAF and 90DAF fruits were also used for further functional analysis. GO annotation were analyzed through Plant MetGenMAP tools [41]. KOG and KEGG pathway annotations were performed using Blastall software against the KOG and KEGG databases [42]. These DEGs were also assigned to functional categories using MapMan (Vvnifera_145, http://mapman.gabipd.org/web/guest/mapmanstore)[43]. MISA script was used to identify SSR loci (http://pgrc.ipk-gatersleben.de/misa/misa.html).

### qRT-PCR validation

qRT-PCR was performed to verify the expression patterns revealed by the RNA-seq study. Purified RNA samples were reverse-transcribed using the PrimeScript RT Reagent Kit with gDNA Eraser (Takara, Dalian, China) as per the manufacturer’s protocol. Forty transcripts were randomly selected for the qRT-PCR assay. Gene specific qRT-PCR primers were designed using Primer3 software (http://primer3.ut.ee/) [44] using the 3’ UTR sequence information, while for the genes lacking sequence information from this region, primers were designed to anneal in the coding region (Table 1). qRT-PCR was carried out using an ABI PRISM 7500 real-time PCR system (Applied Biosystems, USA). Each reaction mix was composed of 10μl 2×SYBR Green Master Mix Reagent (Applied Biosystems, USA), 2.0μl cDNA sample, and 400 nM of gene-specific primers in a final volume of 20μl. PCR conditions were: 2 min at 95°C, followed by 40 cycles of denaturation at 95°C for 10 s and annealing at 60°C for 40 s. The relative mRNA level for each gene was calculated using the 2^-ΔΔCT^ formula [45]. A primer pair was also designed for TC81781 (The Institute for Genomic Research, Release 6.0), encoding an actin protein (housekeeping gene). At least three replicates of each cDNA sample were performed for qRT-PCR analysis.

**Table 1:** qRT-PCR primers.

Gene ID	Forward Primer (3‘-5’)	Reverse Primer (5‘-3’)	Predicted function
VIT_02s0025g00360	GCTCATCCTTCCATTGCTCG	TGGCAGACACCTCCTTTTCT	1-aminocyclopropane-1-carboxylate synthase-like
VIT_11s0016g02380	TCTTGGTTTGGAGAAGGGCT	CCCTAACAAGCTCAGGTCGA	1-aminocyclopropane-1-carboxylate oxidase 1-like
VIT_05s0049g00090	GTTGTTCTGAGAGGTGGGGA	CACACCGGATTCCAAACCAG	ethylene receptor 2-like
VIT_08s0040g01730	AGATTTCTGGAAGCAGCCCT	GACCCAACCCAGCTAGTCTT	ethylene-insensitive protein 2-like
VIT_06s0004g01610	GGAGGATGGCAGAATTCACG	CCACAGGTCTATCTTGCCCA	ethylene insensitive 3-like 3 protein-like
VIT_11s0016g05410	CCTGCCGGGGTATAACAGAT	ATTGCAGGCCCTCAAGAGAT	EIN3-binding F-box protein 1-like
VIT_17s0000g02230	CGTCCCTTTGCATCATCTGG	VGGCTTCCCACTTGCTTTTCA	protein TIFY 6B-like
VIT_02s0012g01320	GGTGGTGGTGGAGATTCTGA	TTTGCAGGTTTTCTCCCACG	transcription factor MYC2-like
VIT_01s0010g02750	CAAAGATCTCCGGCGTGAAG	GATCCTCGCTCTTGTCAGGA	lipoxygenase 6, choloroplastic-like
VIT_03s0063g01820	GTCCTCCTCGACTCCATCAG	TCGGAAGGGTCGAGATATGC	allene oxide synthase, chloroplastic-like
VIT_14s0083g00110	CTTCCTCGTTCAAGCTGCTC	GTGAGGTTGTGGGATGGAGA	unkonwn protein
VIT_10s0042g01250	GTGCTGTTAGTATCTGCCGC	TTGACACATCTCCAGCCAGT	regulatory protein NPR3-like
VIT_11s0016g01990	GGCGGTTTTGGGGTATTTGT	AGAGCACCTCCACCATGAAA	regulatory protein NPR1-like
VIT_08s0007g06160	AAACGCCAGACCCTAAGACA	GTGTGAGCTTGATCCTGCTG	transcription factor HBP-1b(c1)-like
VIT_07s0031g01320	CAGATTAGCACGTGGGGAGA	TCAGAAGGTCCAGGTGTTCC	transcription factor TGA1-like
VIT_03s0088g00700	AGTTGGCGTTGGGTCTATGA	CTGTCAATGAACCACTGCCC	basic form of pathogenesis-related protein 1-like
VIT_08s0040g01710	GGATCAACACCCTCCTCCAA	AGAGGCAAGCTTGGAGTGAT	phenylalanine ammonia-lyase-like
VIT_07s0031g01850	TGCTTCAGAACTGGGAGGAG	ATCGAAGCTCCGCATTCAAC	systemin receptor SR160-like
VIT_18s0001g10690	CCTCCACCACAATCCCAGAT	GTGGAGGAGCGAGGAGATAC	BRI1 kinase inhibitor 1-like
VIT_02s0025g03550	ATTGCGGGTTCTCTGTGGTA	ACAATACCCTCAACACCGGT	probable serine/threonine-protein kinase At5g41260-like
VIT_14s0083g01110	CTCCATGACCACCCAAGAGT	TCAAAGATCACAGCTCGGGT	brassinosteroid-6-oxidase
VIT_19s0085g00830	GTTGGTCTGGGCTTTGAAGG	ATTCACACACTCCGGGAAGT	ent-kaur-16-ene synthase, chloroplastic-like
VIT_18s0001g11320	TGTTCGGTATGCGCATGAAG	TTCAGGCTTCCACTCCTCAG	ent-kaurene oxidase, chloroplastic-like
VIT_19s0140g00140	GGAGAACACACAGACCCTCA	AGAAGGAGTTGGCATCAGGT	gibberellin 2-beta-dioxygenase 1-like
VIT_01s0011g05260	GGACAACACCGATCATCTGC	CGTCGAGATCTGCCAAACTG	GAI1
VIT_16s0050g02620	TGATGGTTGTTGTTGCCGTT	CCGAATGTACTCCGACTCCA	abscisic acid receptor PYL8-like
VIT_13s0175g00120	GGAGTTGTCGCTGAACCATC	CGGAAATTGTGGTTGTGGGT	abscisic acid-insensitive 5-like protein 2-like
VIT_07s0031g00620	TAGTTCAGTGCAGCCTTCGA	CACCCTCTTCTTTTGCCCAC	zeaxanthin epoxidase
VIT_19s0093g00550	AAACTGCTCTCTCCACTCCC	ATTGCGGTGAGACAGAGGAA	9-cis-epoxycarotenoid dioxygenase NCED1, chloroplastic-like
VIT_07s0104g00270	GTGGAGAAGGGAATGGTGGA	TCTTCGTCCAGGAAACCCTC	adenylate isopentenyltransferase 3, chloroplastic-like
VIT_04s0008g01880	GACGAGGTTTGTGGAGAGGA	CCATCCATCCATCGTCCTCA	cytokinin dehydrogenase 7-like
VIT_05s0020g02210	ATGGAGGTGGTTCAGATGCA	CAGGGTTGCTCTCATCTTGC	histidine-containing phosphotransfer protein 1-like
VIT_01s0011g05830	TCGGTGTCATCTTGCTCAGT	AGAGCCCTGGATATCGCAAA	two-component response regulator ARR2-like
VIT_17s0000g07580	CTGACTCGCATTGACAGGTG	CCTCTCCCTCTTCCTCTCCT	two-component response regulator ARR5-like
VIT_13s0067g00330	TGGACAAGAGGACATGGACC	CAGGGCTGCAATGGTCAAAT	auxin transporter-like protein 2
VIT_09s0002g05150	AACCCTAGCTCTACCTCCCA	CCAGCAAGGTGGTTTGAGTC	auxin-induced protein 22A-like
VIT_01s0244g00150	GTTGAGGAGGCATGCTGATG	ATCGCCACTCATTTCCATGC	auxin response factor 2-like
VIT_18s0001g02610	GAGATTGTACGGCGTTGGAC	CAAGTGGTACCAGCTCTCCA	caffeic acid 3-O-methyltransferase-like
VIT_07s0104g01250	AGCATCAGAGGCAGTGAAGT	CATGAGCCTTGCAAACCCAT	flavin-containing monooxygenase YUCCA10-like
VIT_17s0000g08990	GAGATCCAGCGTCGTGAAAC	AGAGACAGACGTTGAAGGCA	tryptophan aminotransferase-related protein 2-like

### Accession codes

The RNA-seq data have been deposited into the NCBI GEO database (https://www.ncbi.nlm.nih.gov/geo/) under the accession number GSE77218. GEO datasets were also downloaded for the further analysis from NCBI GEO database with GEO numbers GSE20511, GSE24561, GSE28779, GSE35172, GSE41206, GSE62744, and GSE76256.

## Results

### Global analysis of RNA-seq data

**Fig 1:**
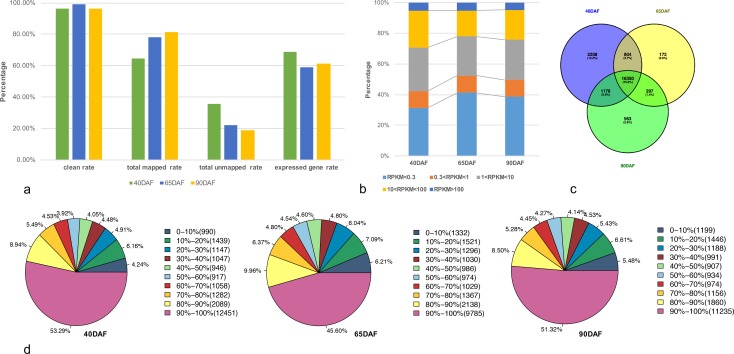
Analysis of global gene expression of RNA-seq data. (a) Summary of RNA-seq reads mapped results in samples (see S1 Table online). The y axis measures the reads or genes mapped percentage. (b) Percentage of genes expressed in samples (see S2 Table online). (c) Venn diagrams showing the number of commonly and uniquely expressed genes among three samples (see S3 Table online). Expressed gene (RPKM > 0.3) in each sample was combined for the analysis. (d) Gene coverage areas in three sample. 40DAF, 65DAF, and 90 DAF were listed as follow. The percentage range are represented by the mapped reads coverage range in each gene. The number in the round brackets are represented the gene number. The percentage are represented the percent of matched genes in the total genes.

RNA-seq data was generated from various developmental stages of grape berry: expanding (40DAF), véraison (65DAF), and ripe (90DAF) fruits. After filtering, the total number of paired-end clean reads in sequencing libraries was 28.8 million, 27.9 million, and 25.5 million respectively in the above mentioned stages. Among these reads, 64.37% to 81.22% were mapped to the genes, while 18.78% to 35.63% of the reads could not be mapped. Genes with normalized expression values lower than 0.3 RPKM (Reads Per Kilobase per Million) were considered as “too low expressed” [46]. Only 58.80% to 68.53% genes were found to be expressed (29,971 genes in total; Fig 1A, S1 Table). The sequencing depth in each sample was larger than 12×, and the expression values of nearly half of the expressed genes were greater than 1 (Fig 1B, S1 and S2 Tables). These results indicated a good representation of the grapevine fruit RNA-seq data. Expressed genes from the three stages of fruits were also combined for analysis. A Venn diagram was used to reveal unique or commonly expressed genes in these three samples (Fig 1C, S3 Table). A total of 16,350 genes were commonly expressed in all samples, while there were 2,208 40DAF sample-specific genes, 172 65DAF sample-specific genes, and 563 90DAF sample-specific genes (Fig 1C, S3 Table). Among these samples, nearly 60% of the expressed genes accounted for over 80% of the full sequence coverage by mapped reads (Fig 1D).

### Gene function analysis and the identification of differentially expressed genes (DEGs)

**Fig 2:**
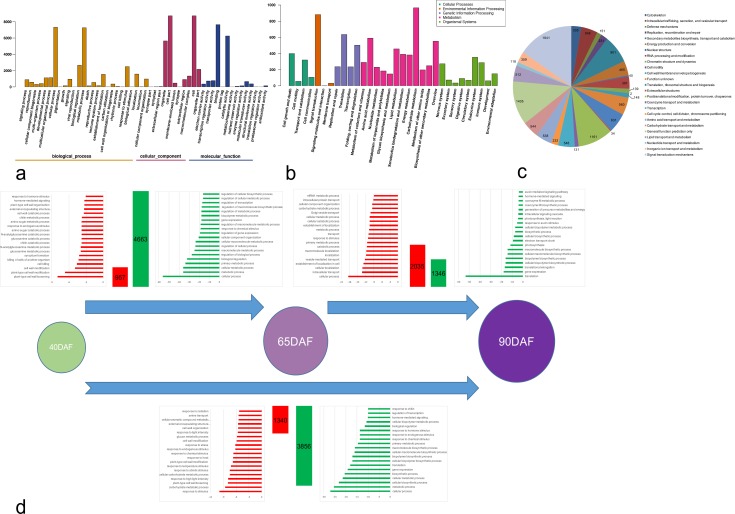
Function analysis of expressed genes. (a) GO analysis of expressed genes. (b) Pathway analysis of expressed genes. (c) KOG analysis of expressed genes. (d) GO analysis of differentially expressed genes in each comparison (Biological Process class).

Gene ontology (GO), Kyoto encyclopedia of genes and genomes (KEGG), and eukaryotic orthologous groups (KOG) analysis were carried out for the expressed genes. GO analysis results indicated that the GO terms “metabolic process” (GO: 0008152, 11,076 transcripts) and “cellular process” (GO: 0009987, 9,879 transcripts) were predominant in the category of biological process. Among the molecular function genes, the two main groups comprised the GO terms “binding” (GO: 0005488, 11,542 transcripts) and “catalytic activity” (GO: 0044464, 9,520 transcripts). The GO terms “cell” (GO: 0005623, 9,336 transcripts) and “cell part” (GO: 0044464, 9,334 transcripts) were the most common categories in the cellular component (Fig 2A).

We classified the KEGG analysis results into five groups: cellular processes, environmental information processing, genetic information processing, metabolism and organismal systems. “Cell growth and death” and “transport and catabolism” were enriched in the first group, “signal transduction” was enriched in the second group, “translation” and “folding, sorting and degradation” were enriched in the third group, “carbohydrate metabolism”, “amino acid metabolism”, and “overview” were enriched in the fourth group, while “nervous system”, “endocrine system”, “immune system” were enriched in the last group (Fig 2B).

KOG (Eukaryotic Orthologous Groups) is another form of COG (Clusters of Orthologous Groups) unique to eukaryotes [47]. The transcripts obtained in our study were compared with the KOG database and were classified into 25 categories. Majority of the transcripts belonged to “signal transduction mechanisms” (1,841), followed by general “function prediction only” (1,405), “posttranslational modification, protein turnover, chaperones” (1,161) and finally “secondary metabolites biosynthesis, transport and catabolism” (901) (Fig 2C, S4 Table).

By using MapMan functional categories, these DEGs were found to cover many functions, with the categories “cell wall”, “lipids”, “secondary metabolism”, “starch-sugar metabolism”, and “light reactions “accounting for the top five categories (S1 Fig). Interestingly, DEGs in “cell wall”, “lipids”, “sugar”, and “secondary metabolism” categories were mainly down-regulated in fruits of 65DAF, and up-regulated in those of 90DAF, while genes in the light reactions category were down-regulated in fruits of both 65DAF and 90DAF.

One of the primary goals of transcriptome sequencing was to compare the levels of gene expression in different samples. In this study, we found that 5,620 transcripts were significantly differentially expressed in DAF65/DAF40 (|log_2_ fold-change (log_2_FC)| ≥ 1 and false discovery rate (FDR) < 0.001), including 957 (17.03%) up-regulated and 4,663 (82.97%) down-regulated transcripts (Fig 2D, S5 Table). A total of 3,381 transcripts were significantly differentially expressed in DAF90/DAF65, including 2,035 (60.19%) up-regulated transcripts and 1,346 (39.81%) down-regulated transcripts (Fig 2D, S6 Table). Out of 5,196 significantly differentially expressed transcripts in DAF90/DAF40, 1,340 (25.79%) genes were up-regulated and 3,856 (74.21%) were down-regulated (Fig 2D, S7 Table). We also performed the GO term enrichment analysis of DEGs between pairs of samples. Among the down-regulated and up-regulated DEGs between 65DAF and 40DAF fruits, 547 and 20 GO terms were respectively over-expressed in the functional process category (S8 Table). Interestingly, at least seven GO terms related to cell wall development were enriched in the GO terms comprising up-regulated DEGs between 65DAF and 40 DAF fruits (Fig 2D, S8 Table). These significantly up-regulated genes were mostly related to cell wall development and cause cell wall loosening, thereby facilitating grapevine fruit enlargement. Meanwhile, two GO terms relating to hormones (“hormone-mediated signaling” and “response to hormone stimulus”) were also enriched in the up-regulated DEGs (62 hormone related genes were found in 957 up-regulated DEGs), which revealed that phytohormones also play an important role in facilitating a swift change from the fruit expanding stage to the véraison stage during grapevine fruit development (Fig 2D, S8 Table). Of the top 20 enriched GO terms in the up-regulated DEGs of 65DAF and 90DAF fruits, 6 GO terms were related to metabolism and catabolism. These observations suggest a deep and complex relationship between these genes during grape fruit development, such as increase in sugar content, reduction in organic acid context, and change in color (Fig 2D, S8 Table).

Moreover, we also analyzed the enrichment of GO terms in the up-regulated and down-regulated DEGs between 40DAF and 90DAF fruits. While cell wall modification terms (*e*.*g*. cell wall modification and cell wall organization) were found only in the up-regulated DEGs, metabolic and catabolic terms (*e*.*g*. “carbohydrate metabolic process”, “cellular carbohydrate metabolic process”, “cellular bio polymer metabolic process”, and “biopolymer biosynthetic process”) were found in both down-regulated as well as up-regulated DEGs (Fig 2D, S8 Table).

#### Specifically expressed DEGs between different fruits

**Fig 3:**
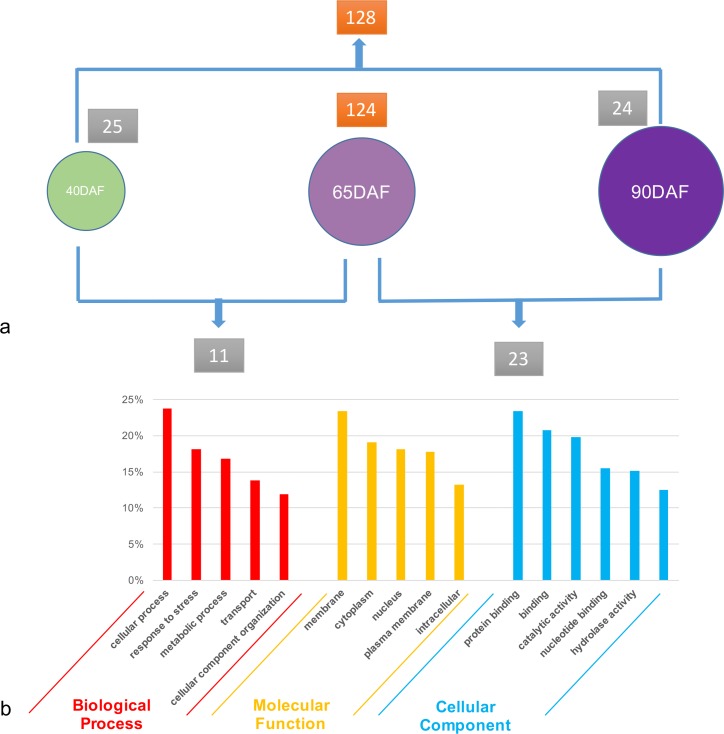
Specially expressed DEGs among these fruits. (a) The number of specially expressed DEGs among these fruits. (b) GO analysis of these DEGs.

On average, nearly 20,000 genes were expressed during each stage of fruit development, and some genes were significantly highly expressed (RPKM value > 100) in one or two stages, while also being less expressed (RPKM value < 10) in other stages. We have also focused on these specifically expressed DEGs among the three stages of fruit development. Interestingly, less than 30 DEGs were found highly expressed in 40DAF fruits, 90DAF fruits, or between 40DAF and 65DAF fruits, and 65DAF and 90DAF fruits. Additionally, more than 100 highly DEGs were found in 65DAF fruits, or between 40DAF and 90DAF fruits (Fig 3A, S9 Table). The five-fold difference between 65 DAF fruits and 40DAF or 90DAF fruits indicated that the véraison (65DAF) stage is very important for grapevine fruit development, and many genes were significantly up- or down-regulated only during this stage. We also used GO analysis to investigate the biological process or molecular function of these specially expressed DEGs among fruits. While most of the greater than 300 DEGs belonged to the “cell process”, “response to stress”, “metabolic process”, “transport”, and “cellular component organization” terms in the biological process class, the remaining DEGs belonged to “protein binding”, “binding”, “catalytic activity”, “nucleotide binding”, and “hydrolase activity” terms in the molecular function class. For cellular component class, “membrane”, “cytoplasm”, “nucleus”, “plasma membrane”, and “intracellular” were the top five GO terms (Fig 3B). Furthermore, for analysis, we mainly focused on: i) genes associated with anthocyanin biosynthesis and transport; ii) transcriptional factors and genes associated with hormone biosynthesis, iii) genes involved in cell wall and lipid metabolism; and iv) genes involved in sugar metabolism.

### DEGs in anthocyanin biosynthesis and transport

**Fig 4:**
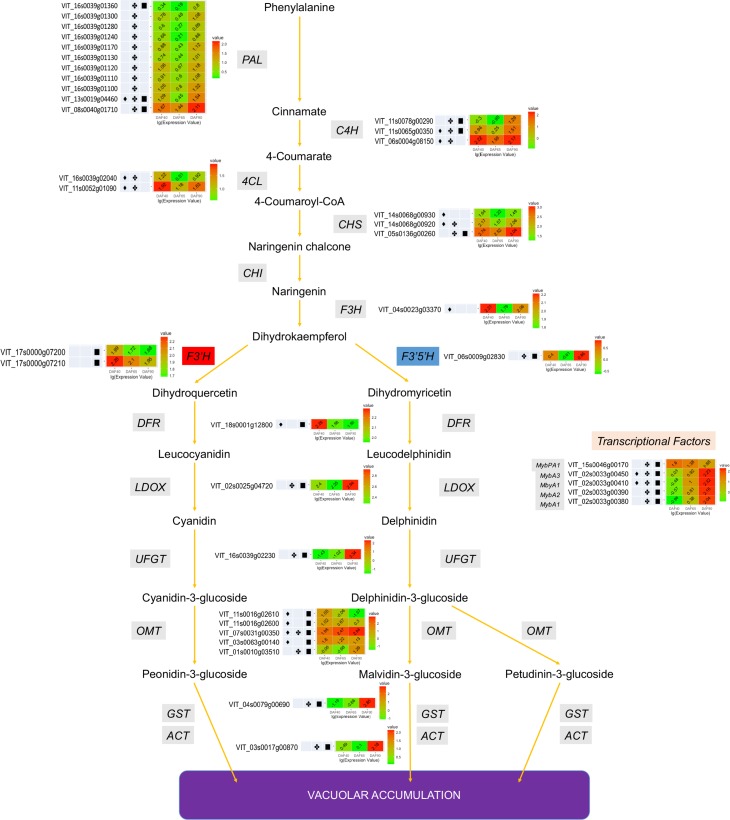
Biological network of anthocyanin biosynthesis and transport pathway in grapevine fruit. ♦, indicated the transcript differential expressed between 65DAF and 40DAF fruits. ✤, indicated the transcript differential expressed between 90DAF and 65DAF fruits. ■, indicated the transcript differential expressed between 90DAF and 40DAF fruits.

The types of anthocyanins found in grape are 3,5-diglucosides of delphinidin, cyanidin, petunidin, peonidin, and malvidin. On the other hand, pelargonidin can barely be identified in grapes [48]. PAL is the first enzyme involved in the phenyl propanoid/anthocyanin biosynthesis pathway. Of the twelve *PALs*, eleven were identified as significantly expressed during grape fruit development, with all of them being down-regulated in 65DAF, and up-regulated in 90DAF (Fig 4, S10 Table). Moreover, *C4H* (three transcripts), *4CL* (two transcripts), and *CHS* (three transcripts) were expressed at a much higher level in 40DAF and 90DAF than in 65DAF. F3H, F3’H, and F3’5’H are key enzymes (branch enzymes) in anthocyanin biosynthesis. When compared with *F3’5’H*, the expression (RPKM value) of *F3Hs* and *F3’Hs* was much higher at each stage. One transcript of *F3H* and two transcripts of *F3’H* were significantly differentially expressed during fruit development, and the expression value was higher than 40 in each stage. Only one transcript of *F3’5’H* was significantly differentially expressed during fruit development, with an expression value of less than 7. The expression values of other *F3’5’H* genes in grapevine were less than 5, and most of them were barely expressed (Fig 4, S10 Table). *DFR*, *LDOX*, *UFGT* are three structural genes of the anthocyanin biosynthesis pathway, and the color of grape fruit is mainly determined by the expression of *UFGT*. In this study, the expression of *DFR* (VIT_18s0001g12800) was found to decrease in 65DAF, and remained constant in 90DAF. The expression level of *LDOX* (VIT_02s0025g04720) decreased in 65DAF, and significantly increased in 90DAF. Interestingly, *UFGT* (VIT_16s0039g02230) was not expressed in 40DAF and 65DAF (0.04 and 0.1 RKPM value), but highly expressed in 90DAF (217.26 RPKM value) (Fig 4, S10 Table). Five transcripts of *OMT*, one transcript of *GST*, and one transcript of *ACT* were also identified as differentially expressed genes in this study. Two transcripts of *OMT* (VIT_07s0031g00350 and VIT_01s0010g03510), one transcript of *GST* (VIT_04s0079g00690), and one transcript of *ACT* (VIT_03s0017g00870) were expressed at much higher levels in 90DAF than in other stages (Fig 4, S10 Table).

Apart from the structural genes, transcriptional factors (TF), especially *Myb* TF, are also involved in the anthocyanin biosynthesis pathway. In this study, two transcripts of *MybA1* (VIT_02s0033g00410 and VIT_02s0033g00380), one transcript of *MybA2* (VIT_02s0033g00390), one transcript of *MybA3* (VIT_02s0033g00450) were barely expressed in 40DAF and 65DAF, and highly expressed in 90DAF. Meanwhile, the expression level of one transcript of *MybPA1* (VIT_15s0046g00170) was found to be decreased in 65DAF and 90DAF (Fig 4, S10 Table).

Presence of anthocyanin pigment is responsible for the red or purple, but not white-green, color of the grapevine berry [49]. In order to compare the difference between the colors of grapevine fruits, we analyzed anthocyanin related-data from seven previous reports (two of them are unpublished, GEO accession number are shown in materials and methods [50–54]; the grape cultivars ‘Norton’, ‘Corvina’, and ‘Muscat Hamburg’ are wine grape types, while ‘Danfeng-2’ (V*itis quinquangularis*) is a table type cultivar. The differences in grapevine fruit color were due to differences in expression of anthocyanin biosynthesis related genes. The fruit skin of most of the wine grapes (*e*.*g*. ‘Cabernet Sauvignon’) was more intense blue than the table grape (*e*.*g*. ‘Fujiminori’) [‘Danfeng-2’ skin color was red]. The expression profiles of the microarray or RNA-seq experiments mentioned above have indicated that some of the transcripts of *F3’5’H* were significantly differentially expressed (also highly expressed) in wine grape cultivars, while barely being expressed in the red colored ‘Danfeng-2’ cultivar. The expression profile of other genes was similar in these cultivars (S11 Table).

### Transcriptional factor-related DEGs

A large number of differentially expressed genes encoding transcriptional factors were identified in this study (S12 Table). In total, 336 (5.98% of total DEGs) and 146 (4.32% of total DEGs) DEGs from 65DAF/40DAF and 90DAF/65DAF respectively were categorized into more than 40 distinct transcription factor families (S12 Table). Between 65DAF and 40DAF fruits, 47 genes encoding transcription factors were found to be up-regulated while 289 were down-regulated. Between 90DAF and 65DAF fruits, 82 genes encoding transcription factors were found to be up-regulated and 64 were down-regulated. Majority of the transcription factor-encoding DEGs were members of the ERF family, followed by MYB, bHLH, WRKY, C3H, and MYB-related families. In case of the ERF family, 29 transcripts were down-regulated between 65DAF and 40DAF fruits, whereas 13 transcripts were down-regulated between 90DAF and 65DAF fruits. In the bHLH family of DEGs, in contrast to up-regulated transcripts, a higher number of down-regulated transcripts was observed between 65DAF and 40DAF or 90DAF and 65DAF. DEGs belonging to the MYB, WRKY, C3H, and MYB-related families were mostly repressed between 65DAF and 40DAF and induced between 90DAF and 65DAF.

### DEGs in cell wall and lipid metabolism

**Fig 5:**
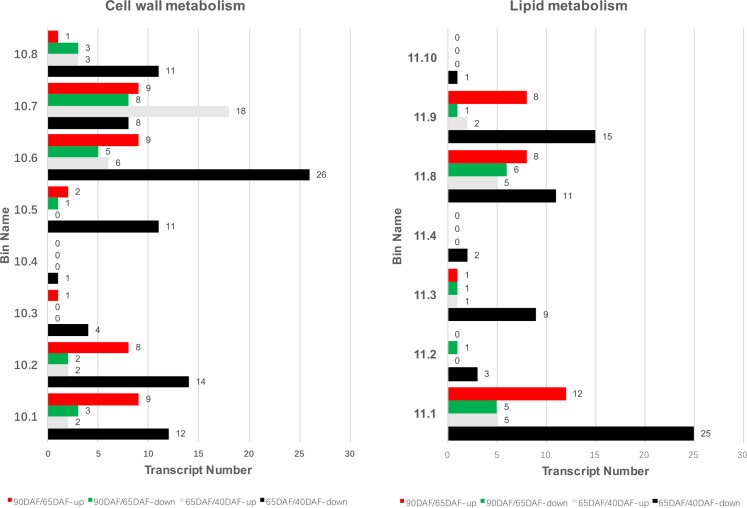
Bin map of cell wall (left) and lipid (right) metabolism between 65DAF and 40DAF, or 90DAF and 60DAF fruits. Note: 10.1, Precursor synthesis; 10.2, Cellulose synthesis; 10.3, Hemicellulose synthesis; 10.4, Pectin synthesis; 10.5, Cell wall proteins; 10.6, Degradation; 10.7, Modification; 10.8, Pectinesterases. 11.1, FA synthesis and FA elongation; 11.2, FA desaturation; 11.3, Phospholipid synthesis; 11.4, TAG synthesis; 11.8, Exotics'(steroids, squalene etc); 11.9, Lipid degradation; 11.10, Glycolipid synthesis.

Eight and seven DEGs enriched cell wall and lipid metabolism related categories were found during grapevine berry development using MapMan (Fig 5). Most DEGs belonging to cell wall precursor, cellulose, hemicellulose and pectin synthesis were down-regulated in 65DAF/40DAF fruits, and up-regulated in 90DAF/65DAF fruits. All DEGs belonging to cell wall proteins in 65DAF/40DAF fruits were down-regulated, and in 90DAF/65DAF fruits only one and two DEGs were down-regulated and up-regulated respectively. Twenty-six DEGs involved in cell wall degradation category were down-regulated in 65DAF/40DAF fruits, while six DEGs were up-regulated. Five DEGs were down-regulated in 90DAF/65DAF fruits, while nine DEGs were up-regulated. Cell wall modification is also an important process of cell wall development. In this study, eighteen up-regulated DEGs and eight down-regulated DEGs were found in 65DAF/40DAF fruits, whereas nine up-regulated DEGs and eight down-regulated DEGs were found in 90DAF/65DAF fruits. Among the pectinesterase related DEGs, eleven were found to be down-regulated and three were up-regulated in 65DAF/40DAF fruits, while three were down-regulated and one was up-regulated in 90DAF/65DAF fruits. Most *cellulose synthase* transcripts were down-regulated in 65DAF/40DAF but up-regulated in 90DAF/65DAF. In contrast, most *expansin* transcripts were up-regulated in 65DAF/40DAF but down-regulated in 90DAF/65DAF. Eight out of fourteen *xyloglucan endotransglucosylase* transcripts were up-regulated in 65DAF/40DAF, and seven out of ten transcripts were up-regulated in 90DAF/65DAF (Fig 5, S5 and S6 Tables).

Fatty acid (FA) synthesis and elongation, FA desaturation, phospholipid synthesis, triacylglycerol (TAG) synthesis, exotics’ (steroids, squalene, *etc*.), lipid degradation, glycolipid synthesis are the main categories of lipid metabolism in grapevine fruits (Fig 5). Most of the DEGs were found to be involved in FA synthesis and elongation, phospholipid synthesis, exotics (steroids, squalene, *etc*.), and lipid degradation categories. Twenty-five DEGs were down-regulated in FA synthesis and elongation category of 65DAF/40DAF fruits, whereas eight DEGs were up-regulated in FA synthesis and elongation category of 90DAF/65DAF fruits. Nine DEGs of the phospholipid synthesis category were down-regulated in 65DAF/40DAF fruits, whereas only one DEG was found in others. Several DEGs of exotics (steroids, squalene, *etc*.) and lipid degradation were repressed in 65DAF/40DAF fruits, while the opposite pattern was observed in 90DAF/65DAF fruits.

### DEGs in starch-sucrose metabolism

DEGs involved in starch-sucrose metabolism are classified into four categories: sucrose synthesis, starch synthesis, sucrose degradation and starch degradation (S2 Fig). In case of the sucrose synthesis and degradation categories, nine DEGs involving sucrose degradation category were down-regulated in 65DAF/40DAF fruits, while few DEGs were found in others. In case of starch synthesis and degradation categories, nine and eight DEGs involved in starch synthesis and starch degradation categories respectively were found in 65DAF/40DAF fruits. Seven DEGs in 90DAF/65DAF fruits were found to be involved in both starch synthesis and starch degradation categories.

### Real time RT-PCR validation

**Fig 6:**
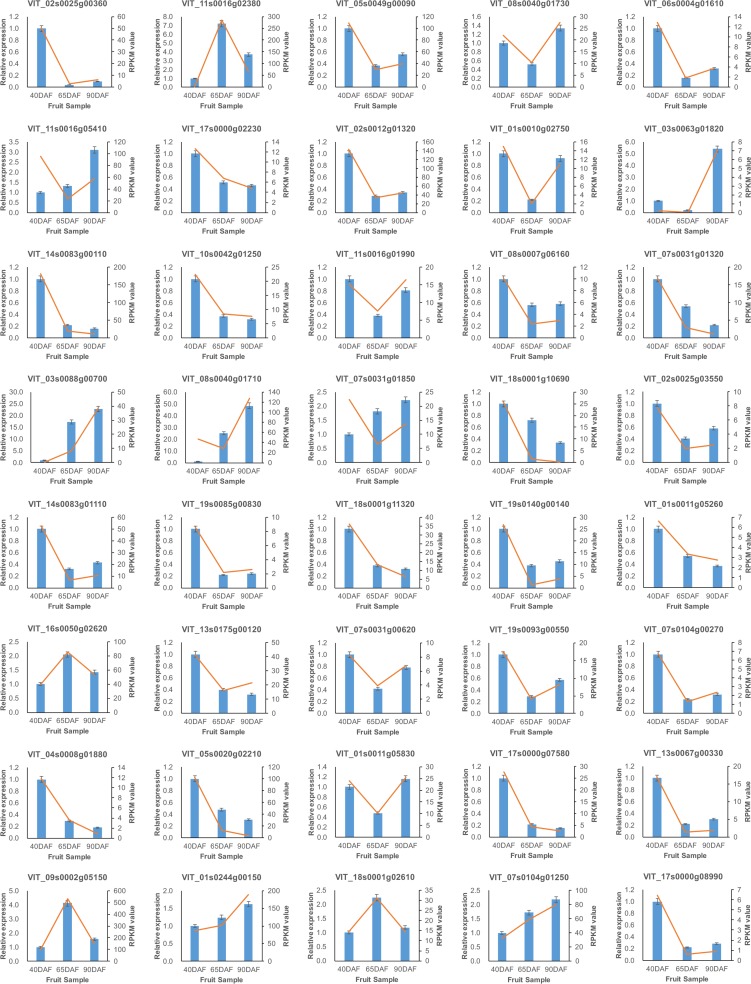
Real time-qPCR validation of differentially expressed transcripts from RNA-seq. Blue bar indicate the RT-qPCR result. Red line indicates the RNA-seq expression data.

qRT-PCR was performed on 40 randomly selected genes using gene-specific primers. Transcript abundance patterns were calculated over the entire course of berry development. qRT-PCR analysis showed an overall agreement of 90% indicating similar trends of transcript abundance when assessed by real-time RT-PCR. The expression profiles of only four genes (VIT_07s0031g01850, VIT_13s0175g00120, VIT_08s0040g01710 and VIT_11s0016g05410) were different from that observed in RNA-seq (Fig 6).

### SSR markers development for phylogenetic analysis in grapevine

**Fig 7:**
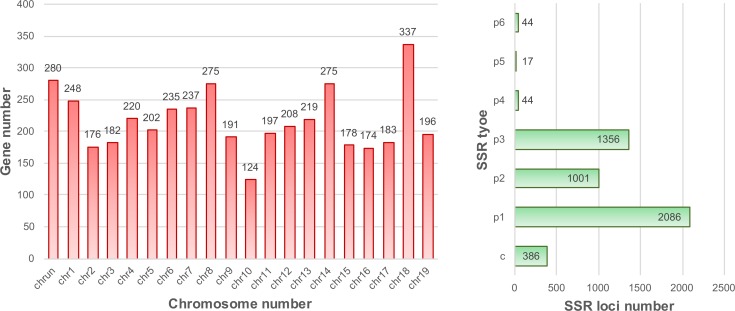
Grapevine SSR distribution (left) and classification (right).

SSR (Simple Sequence Repeat) markers have gradually emerged as preferred molecular markers for many applications in genetics and genomics throughout the genome [55, 56]. Because of improvements in Next Generation Sequencing technologies, the development of SSR markers for application has become much easier [55, 57]. In this study, 4,934 SSR loci were identified from 4,337 genes (Fig 7, S13 Table) that can help in the development of phylogenetic analysis of grapevine and other fruit trees. Among grapevine chromosomes (chromosome 1–19 and unknown), chromosomes 18, unknown, 8, 14 and 1 contain most SSR loci. Single nucleotide repeats were the most abundant class of SSRs, accounting for 42.28% of all SSRs, followed by trimers (27.48%), dimers (20.29%), complexes (7.82%), hexamers (0.89%), tetramers (0.89%) and pentamers (0.35%) (Fig 7).

## Discussion

To date, many experiments have focused on grape fruit development using RNA-seq technology or complemented with other technologies [32, 33, 50, 51, 54, 58–60]. In this study, RNA-seq analysis of transcript abundances during berry development enabled us to carry out a global investigation of gene expression at three time-points during table grape maturation. More than 25 million reads were obtained in each sample, and 5,620, 3,381, and 5,196 transcripts were found to be differentially expressed in DAF65/DAF40, DAF90/DAF65, DAF90/DAF40 respectively. Most of the DEGs were found to be involved in cell wall, lipid, starch-sucrose, and secondary metabolism and light reaction categories.

Table grapevine fruits are colorful, with color ranging from white (green or yellow-skin) to red/purple to blue. Anthocyanins were not detected in the berry skin of the white (or green) cultivar ‘Sauvignonasse’ (Tocai friulano’), and barely detected (less than 1 mg/g) in berry skin of pale pigmented cultivars ‘Gewürztraminer’ and ‘Pinot gris’ [61]. The expression level of *UFGT*, a gene strongly correlated with anthocyanin content, was undetectable in ‘Sauvignonasse’ and barely detectable in the pale colored cultivars 'Pinot gris' and 'Gewürztraminer'. The expression pattern of another important gene in anthocyanin metabolism, *GST*, was similar to that of *UFGT* [61]. Apart from the structural genes, several transcriptional factors (TFs, especially *Mybs*), also play an important role in anthocyanin biosynthesis pathway by controlling the expression of *UFGT* [9, 62]. In the current study, RNA-seq profiles indicated that the expression levels of *UFGT* and *Mybs* were similar in the red/purple or blue colored grapevine fruits (Fig 4, S10 Table), and were higher in the skins of ‘Grignolino’, ‘Moscato rosa’, ‘Nebbiolo’, ‘Pinot noir’ (red skin), and ‘Aglianico’ and ‘Tempranillo’ (dark skin) berries [61].

Reddish color of fruits is determined by cyanidin-based anthocyanins; while purple to blue color is determined by delphinidin-based anthocyanins [63]. In general, the relative proportion of cyanidin- and delphinidin-based anthocyanin pigments determines the final fruit color. Biosynthesis of these two types of anthocyanin pigments is controlled by *F3’H* and *F3’5’H*. By comparing the results of this study (an early ripening table grapevine cultivar) with those of previous studies (some wine grapevines), the expression profiles of *F3’H* transcripts were found to be similar, but the expression profiles of *‘F3’5’H’* transcripts were significantly different. The red skin colored-berries (‘Fujiminori’ and Danfeng-2’) have a weak or barely detectable expression of *F3’5’H* whereas blue skin colored-berries (‘Cabernet Sauvignon’, ‘Sangiovese’, ‘Muscat Hamburg’, etc.) strongly express *F3’5’H* (Fig 4, S10 and S11 Tables). Plants such as rose and carnation are unable to generate purple or blue flowers due to lack of F3’5’H activity [64]. When *F3’5’H* was introduced into plants that naturally do not possess F3’5’H activity, these plants produced purple or blue flowers [65–68]. The skin color of grapes is determined by the quantity and composition of anthocyanins. Previous reports have indicated that the transcription factors (*MybA1a/MybA1b/MybA1c*) regulate anthocyanin biosynthesis in grapevine [9, 13, 14, 69]. Results of this study have enhanced our understanding of the regulatory mechanism of grape fruit skin color. *MybA1* regulated the presence (dark) or absence (white) of fruit skin color, while *F3’H*/*F3’5’H* may regulate the actual color of the fruit (red or purple or blue). The function of *F3’5’H* in table grapevine fruit skin color formation still needs to verified and elaborated by transgenic methods in future. Furthermore, the role of DNA methylation in grape fruit skin color formation also need to be explored.

Fruit softening is caused by dissolution of the middle lamella and disruption of the primary cell wall [70]. Two major modifications of specific polysaccharides occur during grapevine fruit ripening: a dramatic decrease in the type-I arabinogalactan (AGI) of the pectic polysaccharide fraction and an increase in the solubility of galacturonan [16, 71]. We found *beta-galactosidase 1* (*VvBG1*, VIT_18s0001g13230) to be down-regulated in 65DAF/40DAF fruits (Fig 5, S5 Table), which is in agreement with the Northern blot results of Nunan et al. [19]. Repressed *VvBG1* expression before maturation stage is also consistent with the decrease in cell wall galactan in developing grapevine fruits. This also corresponds well to the beta-galactosidase activity accumulation, which is probably important in the hydrolysis of cell wall galactan in grapevine fruit development [16, 71].

The key polymers (pectins, and polygalacturonases) for cell wall strength are pectin-modifying enzymes, which are involved in fruit softening process [72]. In this study, several polygalacturonases (PGs) were identified as DEGs throughout grapevine fruit development, and a representative PG, *VvPG1* (VIT_08s0007g08330), was found to rapidly increase during this process (Fig 5, S5 and S6 Tables). Interestingly, only this gene was extremely up-regulated in the flesh, but was expressed at a much lower level in skin [19, 54, 73]. In the absence of PGs, other pectin modifying-related enzymes have been shown to be required to facilitate pectin breakdown [74]. Fourteen and four *pectinesterase* family genes were isolated in 65DAF/40DAF and 90DAF/65DAF fruits, respectively (Fig 5, S5 and S6 Tables). Most of these transcripts were up-regulated during grapevine development. Some transcripts (*e*.*g*. VIT_07s0005g00730, VIT_11s0016g00300, VIT_04s0044g01010, and VIT_05s0020g01110) were also identified in ‘Muscat Hamburg’, and showed a similar pattern of expression during grapevine fruit development [54].

Other gene families, such as *expansin*, *cellulose synthase* and *xyloglucan endotransglucosylase*, are also important for grapevine fruit softening. Lijavetzky et al. [54] found that six *cellulose synthase* transcripts were down-regulated and six *xyloglucan endotransglucosylase* transcripts were up-regulated in fruits approaching maturity. In our study, while some transcripts have a similar expression pattern as that observed in Lijavetzky's study, some transcripts have the opposite expression pattern (Fig 5, S5 and S6 Tables). The role of such gene families in grapevine fruit softening is uncertain, and may be involved in cell wall extensibility and growth [72, 75]. Consequently, the detailed mechanism of grapevine fruit softening and cracking, and the role of each cell wall modifying gene in the respective processes remains to be uncovered.

Lipid metabolism results indicated that the transcripts for FA synthesis and elongation were repressed throughout grapevine fruit development, and transcripts for lipid degradation were down-regulated in 65DAF/40DAF, but up-regulated in 90DAF/65DAF (Fig 5, S4 and S5 Tables). Most transcripts of enzymes involved in fatty acid metabolism were also found to be down-regulated during fruit development [54].

Previous reports have indicated that IAAs (Indole-3-acetic acid) can suppress or delay the grapevine fruit ripening process, although the role of endogenous IAAs is not clear [76]. A total of 12 IAA biosynthetic genes were identified from the RNA-seq data, of which eight DEGs were up-regulated and four were down-regulated. Normally, the concentration of IAAs has been generally accepted to peak after anthesis and then decline to a very low level in the ripe fruit [77]. Interestingly, all the identified IAAs (13) were found to be down-regulated in our analyses. Most of the ARFs were also found to be down-regulated. A similar expression profile of IAAs and ARFs was also found by Fortes et al.[51]. We hypothesize that IAA mediated delay in grapevine fruit ripening process is mostly affected by responsive genes than by biosynthetic genes. Both IAA and ARF have been identified as rapidly induced auxin response genes [78].

Ethylene is generally considered to have a role in promoting grape fruit ripening [77]. A reduction in berry size and anthocyanin accumulation was observed upon application of 1-methylcyclopropene (an irreversible inhibitor of ethylene receptor) prior to véraison [79]. Furthermore, application of ethylene at véraison led to an increase in berry size and modulated the expression of genes related to ripening [80]. In this study, the expression level of one ACC synthase was found to be high before véraison, and gradually decreased during fruit development (S5 and S6 Tables). Remarkably, the expression level of one *ACC oxidase* was shown to peak at véraison stage, while others were shown to be low (S5 and S6 Tables). Similar trends in *ACC oxidase* genes were also observed by Fortes et al. [51]. Our results suggest that the peak expression of *ACC oxidase* occurs before véraison but some isoforms of *ACC oxidase* were also found to be decreased at véraison.

Ethylene-insensitive 3 (EIN3) protein is a positive regulator of ethylene response. The nuclear protein EIN3 is a transcription factor that regulates the expression of its immediate target genes such as *ERF1* [81]. In agreement with the findings of Fortes et al. [51], we found that the gene encoding EIN3-binding F-box protein and EIN3 protein was down-regulated at véraison. ERF1 belongs to a large family of APETALA2-domain-containing transcription factors that bind to promoters of many ethylene inducible genes. Furthermore, ERF1 is also involved in JA (Jasmonic acid) mediated gene regulation [82]. A transcriptional cascade mediated by EIN3/EIL and ERF proteins leads to the regulation of ethylene controlled gene expression [81]. Interestingly, one transcript coding ERF1 (VIT_05s0049g00510) was highly expressed at véraison and mature stages, while others (VIT_07s0005g03230, VIT_07s0005g03260, and VIT_14s0081g00730) were not expressed (S5 and S6 Tables). The function of ERF1 (VIT_05s0049g00510) is still unknown, but it is believed to be involved in the hormone regulation of fruit development, especially in non-climacteric fruits. Licausi et al. [83] have also indicated that the AP2/ERF family of transcription factors is involved in grape ripening.

Several studies have indicated that there is an increase in free ABA levels around véraison, and genes encoding a *9-cis-epoxycarotenoid dioxygenase* are up-regulated during ripening [51, 77]. In our study, the expression levels of two genes coding for *9-cis-epoxycarotenoid dioxygenase* were much higher in the mature stage than in the pre-véraison and véraison stages (S9 Table). This enzyme catalyzes the crucial step in ABA biosynthesis suggesting that ABA levels increase following véraison [84]. Gibberellin biosynthetic genes were inactivated in the pericarp from veraison [54]. A transcript encoding an ent-kaurene synthase (VIT_19s0085g00830), an ent-kaurene oxidase (VIT_18s0001g11320), and four encoding gibberellin (GA) 2-oxidases (VIT_19s0140g00140, VIT_10s0003g03490, VIT_05s0077g00520, VIT_19s0140g00120) were down-regulated during fruit ripening. Lijavetzky et al. [54] reported that one *ent-kaurenoic acid oxidase* and three *gibberellin (GA) 20-oxidases* were down-regulated during ripening. These results are consistent with a reduction in the production of bioactive GA within the fruit from the onset of ripening [85] and suggest that this phytohormone is more likely to be involved in the regulation of pre-véraison fruit developmental events. These results indicate that hormones play an important role in grapevine fruit development.

## Conclusion

This work describes a comprehensive analysis of the transcriptome during table grapevine fruit ripening (‘Fujiminori’). RNA-seq results indicated that most significant changes in the processes occurred from the expanding stage to the véraison stage. The expression patterns of *F3’H* and *F3’5’H* were the key determinants of red or blue grapevine fruit skin color. DEGs involved in cell wall development, lipid metabolism, and starch-sugar metabolism were also identified. This study provides a foundation for the investigation of fruit quality, fruit softening and cracking, and skin color formation in grapevine.

## Supporting Information

S1 Table The information about RNA-seq. (XLS)

S2 Table RPKM value distribution of each library. (XLS)

S3 Table The expressed gene ID of each library. (XLS)

S4 Table KOG classification. (XLS)

S5 Table DEGs between 40DAF and 65DAF. (XLS)

S6 Table DEGs between 65DAF and 90DAF. (XLS)

S7 Table DEGs between 40DAF and 90DAF. (XLS)

S8 Table GO enrichment analysis between fruits. Blue term indicates the top 20 enriched terms.
(XLS)

S9 Table Highly expressed DEGs between fruits. (XLS)

S10 Table DEGs involved in anthocyanin metabolism. (XLS)

S11 Table Expression profile of *F3H*, *F3’H*, and *F3’5’H* throughout grapevine fruit development in different studies. EL32, beginning of bunch closure, berries touching (if bunches are tight); EL33, Berries still hard and green; EL34, Berries begin to soften, sugar levels start to increase; EL35, Berries begin to color and enlarge; EL36, Berries with intermediate sugar values; EL37, Berries not quite ripe; EL38, Berries harvest-ripe. P, pre-véraison; V1, 50% véraison; V2, 100% véraison; R1, ripening stage 1, with densities between 110–130 g NaCl/L; R2, ripening stage 2, with densities between 130–150 g NaCl/L. Pea, equal EL31, berries pea-size (7 mm diam.); Touch, equal EL32; Soft, equal EL34; Harv, equal EL38.
(XLS)

S12 Table Up-regulated and down-regulated transcriptional factors between fruits. (XLS)

S13 Table The identification result of SSR loci in grapevine transcripts. c, complex repeat motif; p1, single nucleotide repeat motif; p2, di-nucleotide repeat motif; p3, tri nucleotide repeat motif; p4, hexa-nucleotide repeat motif; p5, tetra-nucleotide repeat motif; p6, penta-nucleotide repeat motif.
(XLS)

S1 Fig MapMan pathway figure of the comparison of 40DAF and 65DAF (left), and 65DAF and 90DAF (right) on metabolism overview categories. Dotted line box indicates significantly enriched bins.
(TIF)

S2 Fig Bin map of starch-sucrose metabolism between 65DAF and 40DAF, or 90DAF and 60DAF fruits. (TIF)
