# Supplementary figures and images for: RNA-Sequencing Reveals Biological Networks during Table Grapevine (‘Fujiminori’) Fruit Development
Source: PLoS One. 2017 Jan 24;12(1):e0170571. doi: 10.1371/journal.pone.0170571 (PMC5261597; doi:10.1371/journal.pone.0170571)

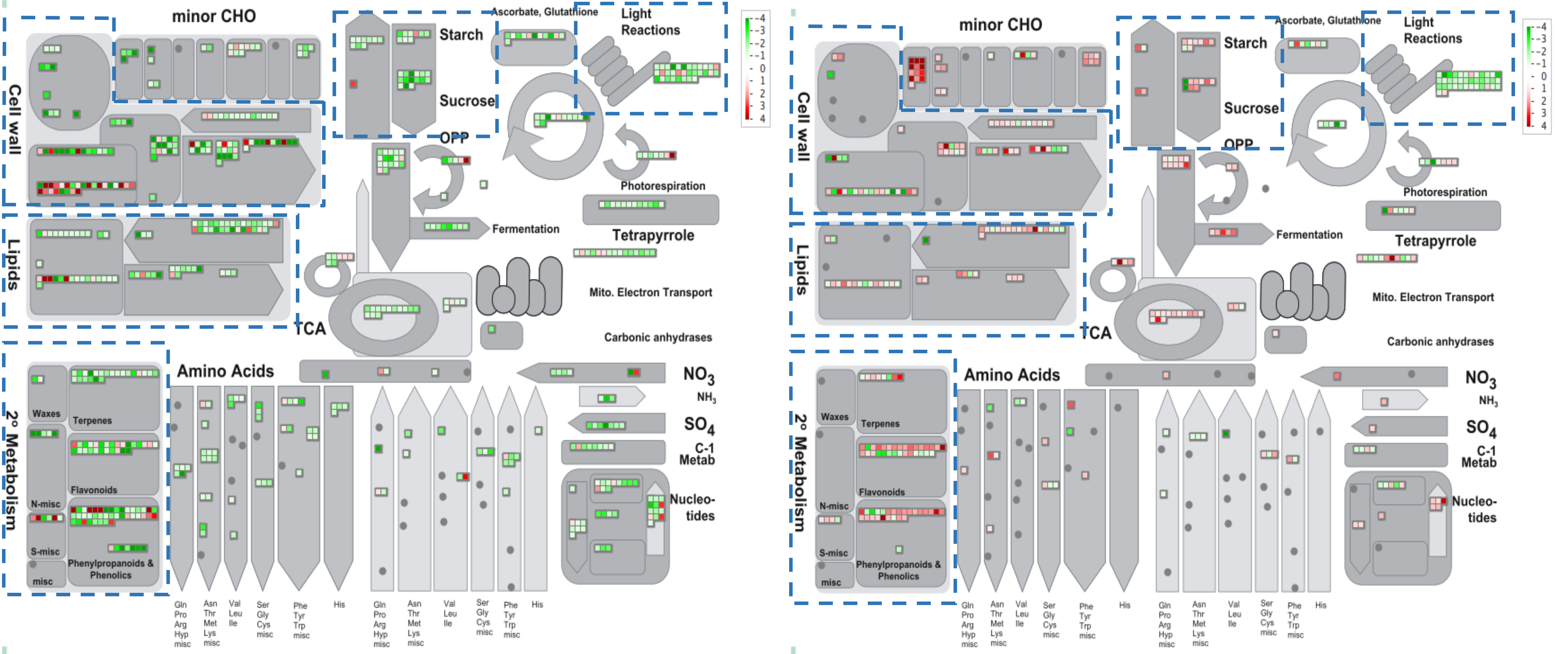

Supplement: S1 Fig — Dotted line box indicates significantly enriched bins. (TIF) [file pone.0170571.s014.tif]

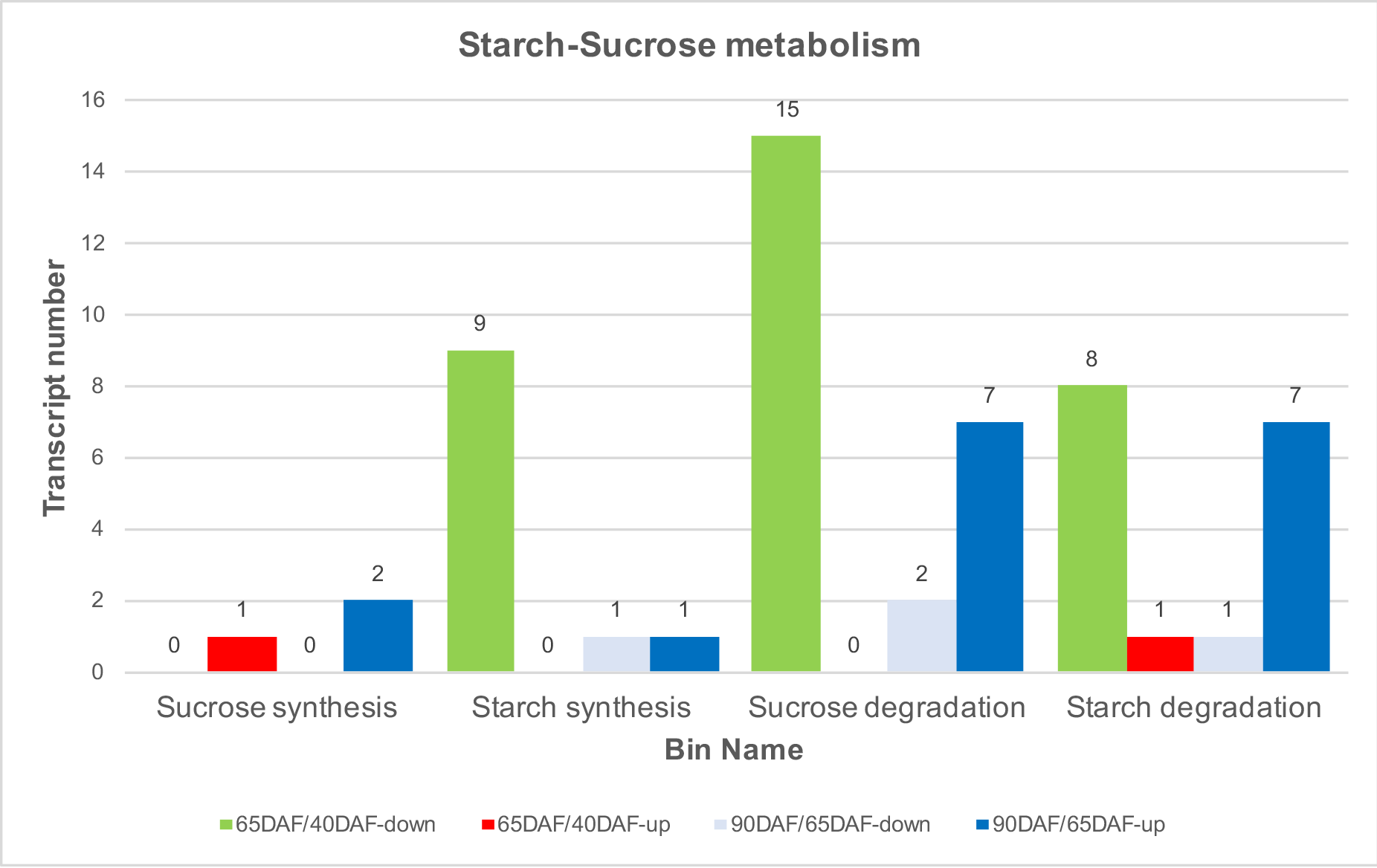

Supplement: S2 Fig — (TIF) [file pone.0170571.s015.tif]
